# Using Weighted Hospital Service Area Networks to Explore Variation in Preventable Hospitalization

**DOI:** 10.1111/1475-6773.12777

**Published:** 2017-09-22

**Authors:** Michael O. Falster, Louisa R. Jorm, Alastair H. Leyland

**Affiliations:** ^1^ Centre for Big Data Research in Health UNSW Sydney Sydney NSW Australia; ^2^ MRC/CSO Social and Public Health Sciences Unit University of Glasgow Glasgow UK

**Keywords:** Patient catchments, multilevel modeling, hospital service areas, preventable hospitalizations

## Abstract

**Objective:**

To demonstrate the use of multiple‐membership multilevel models, which analytically structure patients in a weighted network of hospitals, for exploring between‐hospital variation in preventable hospitalizations.

**Data Sources:**

Cohort of 267,014 people aged over 45 in NSW, Australia.

**Study Design:**

Patterns of patient flow were used to create weighted hospital service area networks (weighted‐HSANs) to 79 large public hospitals of admission. Multiple‐membership multilevel models on rates of preventable hospitalization, modeling participants structured within weighted‐HSANs, were contrasted with models clustering on 72 hospital service areas (HSAs) that assigned participants to a discrete geographic region.

**Data Collection/Extraction Methods:**

Linked survey and hospital admission data.

**Principal Findings:**

Between‐hospital variation in rates of preventable hospitalization was more than two times greater when modeled using weighted‐HSANs rather than HSAs. Use of weighted‐HSANs permitted identification of small hospitals with particularly high rates of admission and influenced performance ranking of hospitals, particularly those with a broadly distributed patient base. There was no significant association with hospital bed occupancy.

**Conclusion:**

Multiple‐membership multilevel models can analytically capture information lost on patient attribution when creating discrete health care catchments. Weighted‐HSANs have broad potential application in health services research and can be used across methods for creating patient catchments.

Both policy makers and health researchers seek to quantify variation in health service use, expenditure, and outcomes. For policy makers, attributing such variation to responsible organizations, such as hospitals or health districts, can create networks of accountability (Fisher et al. 2012) and, through performance monitoring, have the power to drive health care reform (ACSQHC and NHPA 2015). For researchers, quantifying variation allows identification of factors which influence health outcomes, and it can facilitate the development of new metrics for performance and targeted intervention strategies to meet specific health goals.

Central to this process is the ability to analyze data at a level at which variation is meaningful. For example, “preventable” hospitalizations are internationally used as an indicator of access to and quality of primary care (Kruzikas et al. 2004; NHPA 2015), and population variation in preventable hospitalization is often partitioned into geographic “primary care service areas” reflecting natural markets of primary care supply (Mobley et al. 2006; Chang et al. 2011). However, preventable hospitalizations can also be influenced by other health system factors, such as hospitals—which may have a different propensity to admit patients based on factors including the availability of beds (Fisher et al. 2000; Shwartz et al. 2011). This hypothesis remains poorly explored, as such analyses require attributing population variation in admission to the hospital level, and the few studies which have explored these associations (Krakauer et al. 1996; Basu, Friedman, and Burstin 2002; Zhan et al. 2004; Fiorentini et al. 2011; O'Cathain et al. 2013; Berlin et al. 2014) mostly used ecological measures of hospital services at the geographic level. Defining hospitals’ patient catchments to capture hospital‐level variation poses particular difficulties, as patients may not have a designated hospital for admission, administrative data cannot determine a patient's likely hospital where they have not had an admission, and in most health systems choice of hospital is driven not only by geographic proximity but also by provider and patient choice, as well as financial factors such as private health insurance arrangements.

A variety of methods have been developed to create hospital patient catchments, often referred to as “hospital service areas” or HSAs (Wennberg et al. 1999; Klauss et al. 2005). Typically, this involves locating hospitals within geographic regions, then aggregating these regions into larger geographic catchments in which the plurality of patient admissions is to hospitals within the catchment. Alternate methods use different algorithms on patient flows and plurality of residence (Wennberg et al. 1999; Klauss et al. 2005; O'Cathain et al. 2013; Kilaru et al. 2015); spatial analysis on distance to hospitals (Garnick et al. 1987; Epstein 2001; Schuurman et al. 2006); hospital cluster analysis on patterns of patient utilization and geography (Gilmour 2010; Delamater, Shortridge, and Messina 2013b); projected need based on patterns of outpatient service use (Shwartz et al. 2011); or network analyses built on patterns of physician or hospital referrals (Bynum et al. 2007; Landon et al. 2013; Stukel et al. 2013; Casalino et al. 2015).

HSAs are widely used and accepted within health service research and for policy evaluation, and while the purpose of HSAs is often to create clean geographic boundaries of patient catchments for health service planning, most of these methods have the limitation that patient loyalty to the assigned HSA is often quite low, with the HSAs typically capturing between 50 and 80 percent of hospital admissions for their population (Bynum et al. 2007; Stukel et al. 2013; Mazumdar et al. 2014; Kilaru et al. 2015). This is a major conceptual difficulty with HSAs, as they are supposed to represent discrete health care markets, yet patients are receiving care from a variety of additional sources (Bach 2010; Kilaru et al. 2015). Ignoring this can lead to misattribution of variation and potentially bias parameter estimates and statistical inferences (Chung and Beretvas 2012; Leckie 2013). Furthermore, the use of catchments containing multiple hospitals limits the ability to attribute variation to specific hospitals (Shwartz et al. 2011), which may be needed to investigate specific hospital characteristics (rather than geographic aggregates of resources) or to produce hospital performance rankings, such as through league tables (Leyland and Boddy 1998). The use of larger catchments, such as hospital referral regions containing several HSAs and even more facilities, limits the ability to evaluate specific hospital even further (Kilaru et al. 2015).

One method for dealing with such uncertainty is the use of multiple‐membership multilevel models (Browne, Goldstein, and Rasbash 2001; Leckie 2013). Developed within education and social sciences, multiple‐membership multilevel models allow data to be in a hierarchical structure where a lower‐level unit, such as people, can belong to one or more higher‐level units, such as multiple teachers for students in a school, multiple nurses providing care to a patient, or in this case, multiple hospitals servicing a population. While conceptually appealing, the multiple‐membership modeling approach has not been widely utilized in health services research. To the authors’ knowledge, such an approach has not been used in analysis of HSAs, but it could potentially address the major limitations by capturing the uncertainty around patient loyalty and allowing population variation to be correctly attributed to specific hospitals.

In this study, we demonstrate the use of multiple‐membership multilevel models for exploring between‐hospital variation in rates of preventable hospitalization in NSW, Australia. Using these models, we quantify and visualize variation between hospitals in rates of preventable hospitalization and assess their association with a measure of the availability of hospital beds. These results are contrasted to a more traditional approach clustering patients in a single HSA.

## Methods

### Multiple‐Membership Multilevel Models

The general structure of a multilevel model captures the effects of clustering by allowing both regression parameters and error terms to exist at different hierarchical levels. For example, an analysis might wish to look at a variety of person‐level variables (e.g., age, sex, health, education), as well as higher‐level variables of the health system (e.g., type of hospital, bed availability). In general terms, such a model could be expressed as:

$$Y_{ij}=\beta_{0}+\sum_{p=1}^{P}\beta_{p}x_{pi}+\sum_{q=1}^{Q}\beta_{q}x_{qj}+u_{j}+e_{ij}$$

where *I* people are clustered within *J* hospitals or HSAs. *Y*_*ij*_ is the outcome, *x*_*pi*_ are the regression parameters for *P* person‐level variables, and *x*_*qj*_ are the regression parameters for *Q* hospital‐level variables. *β*_0_, *β*_*p*_, and *β*_*q*_ are the regression coefficients for the intercept, person‐level, and hospital‐level parameters accordingly. *e*_*i*_ and *u*_*j*_ represent the random effects at the person and hospital levels, with *e*_*i*_ and *u*_*j*_ belonging to random distributions $e_{ij}∼N\left(0,\sigma_{e}^{2}\right)$ and $u_{j}∼N\left(0,\sigma_{u}^{2}\right)$. Effects for the specific hospitals *u*_*j*_ are estimated from their posterior distributions. Such a model could be extended to generalized linear models for a range of outcomes, such as binary (e.g., whether a patient had a hospital admission) or counts (e.g., number of hospital admissions).

A multiple‐membership multilevel model extends this approach by allowing a weighted structure for each of the hospital‐level components. A linear model with random intercepts can be written as:

$$Y_{ij}=\beta_{0}+\sum_{p=1}^{P}\beta_{p}x_{pi}+\sum_{q=1}^{Q}\sum_{j=1}^{J}w_{j.i}^{\left(2\right)}\beta_{q}x_{qj}^{\left(2\right)}+\sum_{j=1}^{J}w_{j.i}^{\left(2\right)}+u_{j}^{\left(2\right)}+e_{ij}$$

The superscript ^(2)^ in this classification notation (Leckie 2013) indicates model components belonging to the second level of classification (i.e., hospitals), and should further levels be included, these would be indicated by further superscripts ^(3)^, ^(4)^, and so on. Here, $w_{j.i}^{\left(2\right)}$is the probability that person *i* will go to hospital *j* for their admission, with each hospital assigned a weight $\left(0\leq w_{j.i}^{\left(2\right)}\leq 1\right)$ such that the sum of the weights for person i equals 1 $\left(\sum_{j=1}^{J}w_{j.i}^{\left(2\right)}=1\right)$. In this manner, people are proportionately structured within all their potential hospital of admission, and the hospital‐level parameters and random error terms become weighted averages of hospitals in the network. Where people are allocated to a single hospital, this simplifies to the regular two‐level model above.

## Variation in Preventable Hospitalization

### Study Population

Data used for this analysis were obtained from The Sax Institute's 45 and Up Study (Banks et al. 2008), a prospective cohort of 267,014 residents of NSW, Australia, aged 45 and older. Participants were recruited between 2006 and 2009 through the Medicare Australia (Australia's national universal health insurer) enrollment database, where at study entry participants completed a detailed questionnaire containing self‐reported information on their health, sociodemographic characteristics, and risk factor behavior. Participants also provided consent for long‐term follow‐up, including linkage with administrative health datasets.

For each study participant, linked data extracts were obtained for hospital admissions from the NSW Admitted Patient Data Collection (APDC), a census of all hospital separations (discharges, transfers, and deaths) from all NSW public and private hospitals and day‐procedure centers, as well as mortality data from the NSW Registry of Births Death and Marriages (RBDM), which contains fact‐of‐death information on death registrations within Australia. Probabilistic data linkage between datasets was performed by a third party, the NSW Centre for Health Record Linkage (http://www.cherel.org.au/), using Choicemaker software. A manual clerical review on a sample of linked records in the Master Linkage Key found a false‐positive linkage rate of 0.3 percent. Linked hospital data were available for the period 2000–2011 and mortality data from 2006 to 2011.

Participants were excluded if they had an unknown age or area of residence or had inconsistent records possibly indicating incorrect linkage (e.g., death before date of study entry). Ethics approval for the 45 and Up Study was given by the University of New South Wales Human Research Ethics Committee, and ethics approval for this study was given by the NSW Population and Health Services Research Ethics Committee and the University of Western Sydney Research Ethics Committee.

### Hospitalizations and Weighted Hospital Service Area Networks

Records used for this analysis were all hospitalizations during the period of follow‐up, from the date of participants’ study entry (between 2006 and 2009) until death or the end of linked data (31/12/2011), whichever came first. Analyses were restricted to admissions to principal, major, and district public hospitals (peer groups A1‐C2), as private hospitals in Australia have different types of patients with different patterns of care, and smaller facilities such as community hospitals, psychiatric facilities, nursing homes, and rehabilitation centers are often not considered in hospital performance benchmarking (NSW Health 2010; BHI 2015). Changes of type of care within a hospital (e.g., from acute to palliative care), and transfers between hospitals, were considered a continuation of the same episode of care.

Weighted hospital service area networks (weighted‐HSANs) were created using patterns of patient flow for all‐cause hospitalizations. Participants were grouped by their area of residence, in this case postal areas, of which there are over 600 in NSW. Participants were then allocated to all hospitals of admission among participants in their postal area, with the weighting corresponding to the proportional distribution of admissions between hospitals. To assign participants to just a single HSA, all residents of a postal area were allocated to the most common hospital of admission. Not all hospitals had a corresponding HSA population, as some did not provide the plurality of services for any postal area.

As an outcome, a count of all “preventable” hospitalizations for each study participant during their follow‐up period was identified in the hospital claims data according to the definition in the Australian 2012 National Healthcare Agreement (AIHW 2012). The indicator is composed of admissions for 21 conditions, broadly categorized as “chronic,” “acute,” and “vaccine‐preventable” (Table S1), and it is currently used as a high‐level health system performance indicator within Australia.

Hospital data from the same period of time were used for creating weighted‐HSANs and HSAs based on patient flow, and for counting preventable hospitalizations, as catchments defined during a performance evaluation period have been found to better reflect actual patterns of service utilization than a prospective attribution (Lewis et al. 2013).

### Hospital‐ and Person‐Level Characteristics

Hospital bed occupancy rate was identified from hospital benchmarking reports for 2008/2009 (NSW Health 2010), which corresponds to the early period of follow‐up for most study participants. It was calculated as the proportion of occupied bed‐days to the number of available bed‐days for the period and can exceed 100 percent for some hospitals with a high number of same‐day admissions where a single bed is used to treat more than one patient. For models using a weighted‐HSAN, hospital bed occupancy was modeled as a weighted average for all hospitals in the weighted‐HSAN.

Person‐level sociodemographic and health characteristics were obtained from the self‐reported survey completed at entry into the 45 and Up Study, including age, sex, marital status, highest level of education, household income, employment, language spoken at home, health insurance status, number of people that can depend on, body mass index, multimorbidity, number of healthy behaviors, self‐rated health, functional limitation, and psychological distress (Table S2).

### Statistical Analyses

Multilevel Poisson models were used to model “rates” of preventable hospitalization, with counts of the number of hospitalizations per person during follow‐up as the outcome and the log of the follow‐up time as an offset. All models were adjusted for self‐reported personal sociodemographic and health characteristics as fixed effects, so the models were exploring residual variation potentially attributable to the health care system. These variables were included in the analysis as they reflect predisposing, need, and access‐related factors for health service use (Andersen and Newman 1973), and they were previously found to be associated with preventable hospitalization (Falster et al. 2015).

Models were run with hospitals in weighted‐HSANs as the higher‐level units, with between‐hospital variation quantified as the variance of the hospital‐level random‐intercept parameter $\sigma_{u}^{2}$ and as a median rate ratio (MRR) (Larsen and Merlo 2005), such that $MRR=\exp\left(0.95\sqrt{\sigma_{u}^{2}}\right)$. The MRR can be interpreted as the median increase in rate of hospitalization if a person were to move from one hospital to another with a higher rate of hospitalization. To compare this variation to other levels of geographic disaggregation, models were also run with the higher‐level units as either HSAs or statistical local areas (SLAs), another small‐level geographic unit; as well as cross‐classified models with both SLA and HSA, or cross‐classified multiple‐membership models with both SLA and hospitals in a weighted‐HSAN, as the higher‐level units. SLA boundaries are unrelated to postal areas and were analyzed as they are used for indicator performance measurement and evaluation. Postal areas are a smaller geographic unit allowing more granularity in defining HSAs and the weighted‐HSANs.

To rank hospitals with higher‐ or lower‐than‐average rates of admission, median and 95 percent credible intervals were obtained from the posterior distribution of the hospital effects. The ranking of hospitals after adjusting for person‐level characteristics from a model using weighted‐HSANs as the higher‐level unit was compared to rankings from a model using HSAs.

Hospital bed occupancy was subsequently included as a continuous variable, rescaled so that one unit change represents a 10 percent change in hospital bed occupancy, centered on the group mean value. A proportional change in variance (PCV) (Merlo et al. 2006) was used to see how much of the between‐hospital variation was explained by this variable, calculated as the proportional difference between the hospital‐level random‐intercept parameter $\sigma_{u}^{2}$ after including hospital bed occupancy in the model. Changes in model fit were assessed using the deviance information criteria (DIC).

All data preparation was performed in SAS v9.3, and all modeling was performed in MLwiN 2.25, using MCMC estimation with inference based on 20,000 samples following a burn‐in of 5,000. Trajectories of stored parameter estimates were visually checked for irregular distributions and convergence to a unimodal distribution.

## Results

There were 267,014 study participants, of which *n* = 78 were excluded for having unknown age, unknown area of residence in NSW, or incompatible dates in the linked data. The remaining 266,936 participants had an average follow‐up of 3.7 years and resided within 612 different postal areas, each containing between 1 and 4,166 participants. *n* = 82,553 participants (31 percent) had one or more all‐cause hospitalizations to a major public hospital during follow‐up, for a total of *n* = 267,032 admissions to 79 different hospitals. Participants in 19 postal areas did not have any hospitalizations during follow‐up; the 174 participants residing in these areas were excluded, leaving 266,762 in 593 areas for analysis (Table S3).

**Figure 1:**
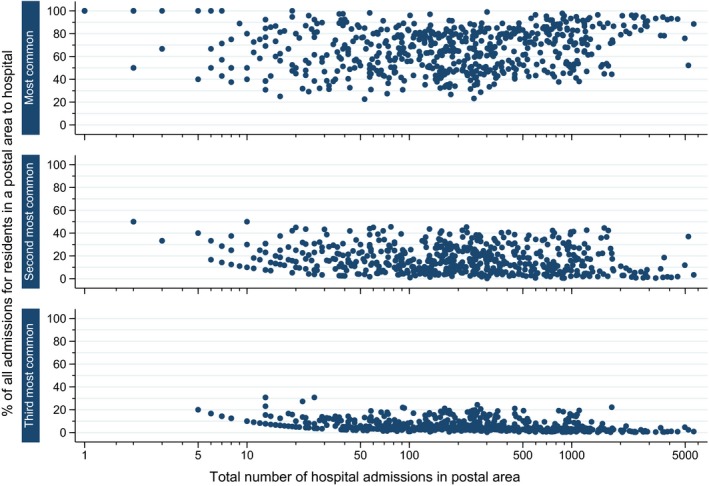
Proportion of All‐Cause Hospitalizations for Study Participants in 593 Postal Areas in NSW, Australia, Which Are to the Most Common, Second Most Common, and Third Most Common Hospitals of Admission [Color figure can be viewed at wileyonlinelibrary.com]

Within each postal area, participants were admitted to a mean of 15 different hospitals (range 1–56), which formed the basis of the weighting for the HSAN (Table 1). Figure 1 shows the proportion of admissions within the 593 postal areas which were to the most common, second most common, or third most common hospital of admission. On average, the most common hospital accounted for 67 percent of admissions in a postal area, although in almost a quarter of postal areas (24 percent) this hospital accounted for no more than half of all admissions. The second most common hospital of admission accounted for an average of 17 percent of admissions in a postal area, although in 11 percent of postal areas it accounted for more than one‐third of all admissions.

**Table 1:** Characteristics of Weighting Structure between Study Participants, Postal Areas, Hospital Service Areas (HSAs), and Weighted Hospital Service Area Networks (Weighted‐HSANs)

	Mean	Interquartile Range	Min–max
Postal areas (*n* = 593)			
Number of study participants	451	82–561	1–4,166
Number of all‐cause hospitalizations	450	63–518	1–5,642
Number of public hospitals of admission	15	8–20	1–56
% all‐cause hospitalizations to the			
Most common hospital	67	51–81	23–100
Second most common hospital	17	7–25	0–50
Third most common hospital	6	2–9	0–31
Hospital service areas (*n* = 72)			
Study patient catchment size	3,705	1160–5798	12–12,801
Postal areas included	8	3–12	1–27
Market share index (%)	69	64 ‐ 87	0–97
Hospitals, from weighted‐HSANs (*n* = 79)			
Weighted study patient catchment size	3,377	973–5720	277–13,227
Total postal areas serviced	111	50–136	17–377
Where hospital weight >5%	19	8–22	1–73
Where hospital weight >10%	14	6–17	0–57
Where hospital weight >20%	10	4–14	0–44
Where hospital weight >50%	6	1–10	0–26
Study participants (*n* = 266,762)			
Number of hospitals in weighted‐HSAN	26	15–34	1–56
% weighting which is to the:			
Most common hospital	70	54–85	23–100
Second most common hospital	14	4–22	0–50
Third most common hospital	5	2–6	0–31

Aggregating postal areas into HSAs based on the most common hospital of admission resulted in 72 HSAs from the total of 79 major hospitals. HSAs were each comprised of a mean of eight postal areas and contained a mean of 3,705 study participants (Table 1). After applying the weighting structure from postal areas to the study population, participants each had a mean of 26 hospitals within their weighted‐HSAN, with the most common hospital of admission accounting for a mean 70.4 percent of the weighting.

**Figure 2:**
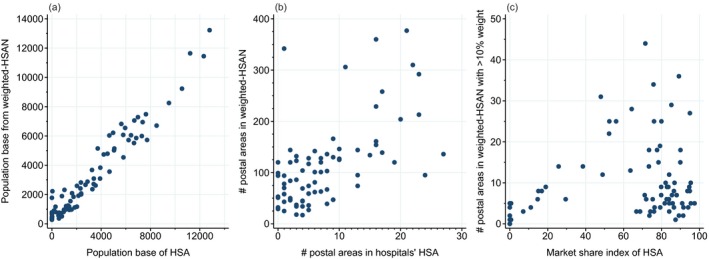
Characteristics of Hospitals When Analyzed Using an HSA or Weighted‐HSAN, Including (a) Population Base, (b) Postal Areas Used to Construct Patient Catchments, and (c) Market Share Index of HSA and Number of Postal Areas Making a Meaningful Contribution to Weighted‐HSAN [Color figure can be viewed at wileyonlinelibrary.com]

There was broad correlation between the size of the population base for HSAs and corresponding hospitals from weighted‐HSAN, although hospitals drew their population from a much larger number of postal areas when using a weighted‐HSAN (Figure 2). While many hospitals had a market share index (the proportion of admissions which are from within their catchment) from their HSA over 70 percent, there were a number of outlying hospitals for which this was poor (Figure 2). Further comparisons of characteristics of hospitals from using HSAs and weighted‐HSANs are in Figure S1.

### Hospital Variation in Preventable Hospitalization

During follow‐up, there were 26,728 preventable hospitalizations among 16,999 (6.3 percent) study participants. After adjusting for personal sociodemographic and health characteristics, there was significant residual variation between hospitals in rates of preventable hospitalizations (Table 2), such that a person moving to another hospital network with a higher rate of admission would face a median 41 percent increase in their rate of hospitalization (MRR: 1.41, 95 percent CIs: 1.31–1.54). The amount of residual variation which sat in models structured on weighted‐HSANs ($\sigma^{2}$=0.130) was over two times as high as models clustered on HSAs ($\sigma^{2}$ = 0.059). However, the variation between SLAs ($\sigma^{2}$ = 0.291) was much greater than for either weighted‐HSANs or HSAs, and models including SLA consequently had a lower DIC (Table S4), possibly due to increased granularity from the larger number of higher‐level units. When people were additionally clustered within SLAs, there were similar amounts of variation between hospitals in a weighted‐HSAN ($\sigma^{2}$ = 0.234) and SLAs ($\sigma^{2}$ = 0.234), but less variation between HSAs ($\sigma^{2}$ = 0.089) than between SLAs ($\sigma^{2}$ = 0.230). These cross‐classified approaches resulted in the lowest DIC (Table S4).

**Table 2:** Random‐Intercept Variance Parameters from Models on Rates of Preventable Hospitalization,^a^ with Higher‐Level Units as Either Hospitals in Weighted Hospital Service Area Networks (Weighted‐HSANs), Hospital Service Areas (HSAs), or Statistical Local Areas (SLAs)

Higher‐Level Unit(s) of Multilevel Model	Variance Estimate (and SE of Variance)		
	Hospitals in Weighted‐HSAN (*n* = 79)	Hospital Service Area (*n* = 72)	Statistical Local Area (*n* = 173)
Weighted hospital service area network^b^	0.130 (0.032)	–	–
Hospital service area (HSA)^c^	–	0.059 (0.012)	–
Statistical local area (SLA)^c^	–	–	0.291 (0.039)
Both weighted‐HSAN and SLA^d^	0.234 (0.061)	–	0.234 (0.061)
Both HSA and SLA^e^	–	0.089 (0.022)	0.230 (0.033)

a Multilevel Poisson models, adjusted for sociodemographic and health characteristics of study participants.

b Two‐level multiple‐membership multilevel model.

c Two‐level multilevel model.

d Three‐level cross‐classified multiple‐membership multilevel model.

e Three‐level cross‐classified multilevel model.

**Figure 3:**
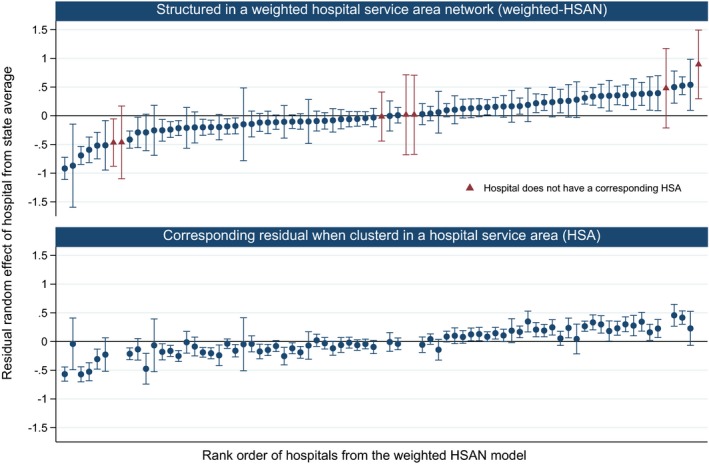
Ranking of Hospitals Based on Estimated Effects on Rates of Preventable Hospitalizations* with Participants Structured within a Weighted‐HSAN, and Corresponding Values with Participants Clustered Using a Single HSA [Color figure can be viewed at wileyonlinelibrary.com] *Note*. *Two‐Level Multilevel Poisson Model, Adjusted for Sociodemographic and Health Characteristics of Study Participants (see Table S2).

There was much variation between hospitals in their effects on hospitalization (Figure 3), with many hospitals having significantly lower‐ or higher‐than‐average rates of admission. The ranking of hospitals using weighted‐HSANs was notably different to the ranking of HSAs based on the primary hospital of admission. For example, one hospital had relatively low rates of preventable hospitalization in the weighted‐HSAN model but average rates when modeled as a HSA. This hospital is an acute facility in a major city (Sydney) with one small postal area forming its HSA (43 people). However, it was the second most common hospital of admission in 19 postal areas and serviced participants from a total of 342 postal areas, although it only accounted for a large proportion of admissions (>10 percent) in four of these. With a weighted‐HSAN patient catchment of 2227 people, the HSA represented only 0.2 percent of this hospital's admissions from the study population.

The weighted‐HSAN model also estimated effects for an additional seven hospitals that did not form the basis for any corresponding HSA (Figure 3). For example, the hospital with the highest effect on preventable hospitalization did not have a corresponding HSA; it was a smaller district hospital within 30 minutes of drive to a large base hospital. Its population base was drawn from 29 postal areas, including four postal areas where it was the second most common hospital of admission and accounted for between 8 and 26 percent of admissions. Conversely, the two additional hospitals in the center of the figure were specialized acute hospitals with small weighted populations (277 and 348 people) drawn from a large number of postal areas (99 and 120, respectively) from which they were at most the third or fourth most common hospital of admission.

### Hospital Bed Occupancy and Preventable Hospitalization

In models structured on a weighted‐HSAN, there was no significant association between a 10 percent increase in hospital bed occupancy rate and preventable hospitalizations (IRR: 1.01, 95 percent CIs: 0.96–1.07). This result was similar when clustering people in the leading hospital of a HSA and was not impacted by the additional clustering of people within an SLA (Table S5).

Across all models, the inclusion of the bed occupancy variable had only minor impacts on the between‐hospital or between‐HSA variation (PCV<2.5 percent) and the model DIC (Tables S4andS6). In contrast, the personal health and sociodemographic factors included in the model had already explained 65 percent and 44 percent of the between‐HSAN and between‐HSA variation accordingly and had major impacts on the DIC (Tables S4andS6). Incidence rate ratios for all person‐level variables were almost identical in analyses using weighted‐HSANs or HSAs (Table S2).

## Discussion

In this study, we demonstrated the novel use of weighted hospital service area networks and multiple‐membership multilevel models to explore how variation in a population‐level outcome could be attributed to hospitals. In our analysis on preventable hospitalization, we found more than twice the variation between hospitals compared to a more traditional approach using HSAs. This variation captures information usually lost through assigning a population to discrete patient catchments. Weighted‐HSANs produced notably different results for some hospitals, such as those with a broadly distributed patient base, and identified smaller hospitals which would otherwise not be considered. Additionally, they allow assessment of specific hospital characteristics (such as bed occupancy), as opposed to the geographically based measures commonly explored. The multiple‐membership models have a clear application within health services research for analyzing variation between hospitals, or other health services without clear patient attribution, particularly as they can be applied using an extension of current popular methodologies.

### Hospital Variation in Preventable Hospitalization

Variation in hospital practice has long been hypothesized to be a contributing factor to the preventable hospitalizations health performance indicator, but there has been almost no quantification of this effect. While one study estimated hospital‐based rates of admission using a projected patient catchment from the distribution of admissions in age‐ and sex‐stratified groups, it was unable to attribute variation to both the patient and hospital levels (O'Cathain et al. 2013).

Using weighted‐HSANs, the current study found no association between hospital bed occupancy and preventable hospitalization. This was surprising, given the well‐established Roemer's law, that is, that the availability of hospital beds leads to higher levels of utilization (Fisher et al. 2000; Shwartz et al. 2011; Delamater et al. 2013a). We used a measure of bed availability attributable to hospitals (occupancy rate), different to the more geographic measures usually explored in the literature (beds per capita), which may explain some of this finding. However, in previous studies exploring preventable hospitalizations and number of beds or inpatient bed‐days per capita, many (Krakauer et al. 1996; Basu, Friedman, and Burstin 2002; Fiorentini et al. 2011; Berlin et al. 2014) but not all (Zhan et al. 2004; O'Cathain et al. 2013) found no significant associations. It may be that a more nuanced exploration of features related to hospital capacity, such as the presence of an emergency department or the role of the hospital in the community, is required, and the weighted‐HSANs method now allows such hypotheses to be explored.

Results from this Australian study may be most comparable to other countries with a universal health care system. Variation was explored between large public hospitals in NSW, which are those used for standardized reporting of hospital performance, but there may be further uncaptured variation between private or smaller community hospitals. Results may also be sensitive to methods used for determining hospital weights. The use of a Poisson model, while common in analyses on preventable hospitalizations, may also fail to adequately capture variation, with residual overdispersion potentially resulting in less accurate variance estimates and CIs.

### Weighted Hospital Service Area Networks

The use of weighted‐HSANs and multiple‐membership multilevel models has broader potential applications in health services research. While multiple‐membership multilevel models have been used on patient populations with known patterns of care, such as patients receiving care from multiple facilities, this approach has not previously been applied to investigate population‐level outcomes where prospective providers of care are unknown. For example, these methods could be used to investigate how availability of regional hospital birthing facilities impacts maternal choices on delivery, whether hospital‐based outpatient services reduce patient admissions, or whether the presence of an emergency department results in differing levels of discretionary hospitalizations.

One advantage of the multiple‐membership modeling approach is the ability to assess exposures and explore residual variation at the hospital level. While HSAs allow this to some extent, their geographic nature and aggregation of facilities limit more detailed exploration. In this analysis, we identified outlying hospitals that would be all but invisible to standard analytic approaches.

Given the range of potential methods for creating patient catchments, results may be sensitive to the choice of method used. Geographically based methods often have the difficulty of allocating both the patient population and the exposure (e.g., number of hospital beds) to distinct geographic regions using the same pattern of patient flows, potentially inducing an artificial correlation (Shwartz et al. 2011). The current analysis partially overcame this difficulty using an exposure already attributed to hospitals (bed occupancy) and an outcome (preventable hospitalizations) different to the services determining the weighting structure (all‐cause hospitalization).

While many HSAs did have a high market share, our analysis also demonstrates that patient catchments for some services may be poorly defined, such as the outlying hospital with a small HSA but a broadly distributed patient base. It was for these facilities that the use of the weighted‐HSANs had the largest impact. While further refinement of HSAs may improve accuracy of some patient catchments, there will remain some regions which are meaningfully serviced by multiple facilities, and some facilities which meaningfully service a broad population. This is a constant limitation of HSAs. Manual revision of HSAs uses considerable resources, such as those constructed for the Dartmouth Atlas (Wennberg et al. 1999), and it is not always practicable for such boundaries to be reconstructed, with many existing boundaries continuing to be used even if the current services underpinning these catchments have changed.

While multiple‐membership multilevel models lose the advantages of a clean and discrete population base, more recent methods for creating HSAs have been moving toward capturing “natural” patterns of health service use for a more robust evaluation of services, as well as automated methods for HSA construction (Hu, Wang, and Xierali 2018), and the use of multiple‐membership models seems a logical evolution. A key strength of the multiple‐membership analytic approach is that it could potentially be used across a range of alternative methods for constructing patient catchments. For example, networks of patients, physicians, and hospital referrals could use patterns of referrals to create a weighting structure, much like the patterns of hospital patient flow used in this analysis. Implementation would require using information on the probabilistic allocation of patients to hospitals from within each respective algorithm, usually produced but discarded following allocation, to create the weighting structure for the hospital networks.

A limitation is that multiple‐membership multilevel models can be complex and currently require specialized multilevel modeling software. There remains limited support for more complex models (e.g., multiple‐membership zero‐inflated Poisson models); however, the capacity of statistical software to manage complex hierarchical structures is improving, and further use of multiple‐membership multilevel models may help facilitate such change. A further limitation is that while the multiple‐membership models use a weighting structure to allocate participants within a hospital network, the outcome (in this case, number of preventable hospitalizations) is not classified according to hospital of admission, although such practice is standard in population‐based analyses on admission rates.

## Conclusion

The needs of researchers and health policy makers to capture service‐level variation and create accountable care organizations must be met with statistical methods that fully utilize available information. The use of weighted hospital service area networks and multiple‐membership multilevel models directly addresses the uncertainty inherent in patient catchments used for analyzing and evaluating hospital performance, and this study found more than two times the variation in preventable hospitalization than a standard approach using hospital service areas. By bringing the analysis back to the level of the hospital, this approach will also enable health researchers to explore associations between population health service use and outcomes with a wider range of hospital characteristics.

## Supporting information

Appendix SA1: Author Matrix.

Table S1: Codes for Identifying Preventable Hospitalizations.
Table S2: Incidence Rate Ratios (IRRs) from Multilevel Models with Higher‐Level Units as Either Hospitals in Weighted Hospital Service Area Networks (Weighted‐HSAN) or Hospital Service Areas (HSAs).
Table S3: Demographic Distribution of Study Participants and Number of Hospitalizations to Major Public Hospitals.
Table S4: Reduction in Deviance Information Criterion (DIC) Statistic From a Single‐Level Age‐ and Sex‐Adjusted Model, Structuring Persons in Various Higher‐Level Units and Sequentially Adjusting for (1) Age and Sex, (2) Further Personal Sociodemographic and Health Characteristics, and (3) Hospital Bed Occupancy.
Table S5: Incidence Rate Ratio (IRR) for Preventable Hospitalization from a 10% Increase in Average Hospital Bed Occupancy Rate, from Models* with Higher‐Level Units as Either Hospitals in Weighted Hospital Service Area Networks (Weighted‐HSAN) or Hospital Service Areas (HSAs), and Statistical Local Areas (SLAs).
Table S6: Proportional Change in Between‐Hospital, HSA, or SLA Level Variance after Sequentially Adjusting for (1) Age and Sex, (2) Further Personal Sociodemographic and Health Characteristics, and (3) Hospital Bed Occupancy.
Figure S1: Correlation between Hospital‐Level Characteristics from Patient Populations Constructed Using a Hospital Service Area (HSA) and Weighted Hospital Service Area Network (Weighted‐HSAN).
Figure S2: Model Specification of Two‐Level Multiple‐Membership Multilevel Model, with Persons Structured within a Weighted Hospital Service Area Network (Weighted‐HSAN), Adjusted for Personal Sociodemographic and Health Characteristics, as Well as Average Hospital Bed Occupancy Rate (Continuous Variable, as a 10% Increase).

## References

[hesr12777-bib-0001] ACSQHC and NHPA . 2015 Australian Atlas of Healthcare Variation. Australian Commission on Safety and Quality in Health Care and National Health Performance Authority, Sydney, NSW.

[hesr12777-bib-0002] AIHW . 2012 “National Healthcare Agreement: PI 22‐Selected Potentially Preventable Hospitalisations, 2012” Australian Institute of Health and Welfare [accessed on May 18, 2012]. Available at http://meteor.aihw.gov.au/content/index.phtml/itemId/443687

[hesr12777-bib-0003] Andersen, R. , and J. F. Newman . 1973 “Societal and Individual Determinants of Medical Care Utilization in the United States.” The Milbank Memorial Fund Quarterly. Health and Society 51 (1): 95–124. 4198894

[hesr12777-bib-0004] Bach, P. B. 2010 “A map to bad Policy–Hospital Efficiency Measures in the Dartmouth Atlas.” New England Journal of Medicine 362 (7): 569–73. 10.1056/NEJMp0909947 PMC3680120 20164483

[hesr12777-bib-0005] Banks, E. , S. Redman , L. Jorm , B. Armstrong , A. Bauman , J. Beard , V. Beral , J. Byles , S. Corbett , R. Cumming , M. Harris , F. Sitas , W. Smith , L. Taylor , S. Wutzke , and S. Lujic . 2008 “Cohort Profile: The 45 and up Study.” International Journal of Epidemiology 37 (5): 941–7. 17881411 10.1093/ije/dym184 PMC2557061

[hesr12777-bib-0006] Basu, J. , B. Friedman , and H. Burstin . 2002 “Primary Care, HMO Enrollment, and Hospitalization for Ambulatory Care Sensitive Conditions: A New Approach.” Medical Care 40 (12): 1260–9. 12458307 10.1097/00005650-200212000-00013

[hesr12777-bib-0007] Berlin, C. , A. Busato , T. Rosemann , S. Djalali , and M. Maessen . 2014 “Avoidable Hospitalizations in Switzerland: A Small Area Analysis on Regional Variation, Density of Physicians, Hospital Supply and Rurality.” BMC Health Services Research 14 (1): 289. 24992827 10.1186/1472-6963-14-289 PMC4091658

[hesr12777-bib-0008] BHI . 2015 Spotlight on Measurement: Return to Acute Care Following Hospitalisation, Spotlight on Readmissions. Sydney, NSW: Bureau of Health Information.

[hesr12777-bib-0009] Browne, W. J. , H. Goldstein , and J. Rasbash . 2001 “Multiple Membership Multiple Classification (MMMC) Models.” Statistical Modelling 1 (2): 103–24.

[hesr12777-bib-0010] Bynum, J. P. , E. Bernal‐Delgado , D. Gottlieb , and E. Fisher . 2007 “Assigning Ambulatory Patients and Their Physicians to Hospitals: A Method for Obtaining Population‐Based Provider Performance Measurements.” Health Services Research 42 (1 Pt 1): 45–62. 17355581 10.1111/j.1475-6773.2006.00633.x PMC1955742

[hesr12777-bib-0011] Casalino, L. P. , M. F. Pesko , A. M. Ryan , D. J. Nyweide , T. J. Iwashyna , X. Sun , J. Mendelsohn , and J. Moody . 2015 “Physician Networks and Ambulatory Care‐Sensitive Admissions.” Medical Care 53 (6): 534–41. 25906013 10.1097/MLR.0000000000000365

[hesr12777-bib-0012] Chang, C. H. , T. A. Stukel , A. B. Flood , and D. C. Goodman . 2011 “Primary Care Physician Workforce and Medicare Beneficiaries’ Health Outcomes.” Journal of the American Medical Association 305 (20): 2096–104. 21610242 10.1001/jama.2011.665 PMC3108147

[hesr12777-bib-0013] Chung, H. , and S. N. Beretvas . 2012 “The Impact of Ignoring Multiple Membership Data Structures in Multilevel Models.” British Journal of Mathematical and Statistical Psychology 65 (2): 185–200. 21732931 10.1111/j.2044-8317.2011.02023.x

[hesr12777-bib-0015] Delamater, P. L. , J. P. Messina , S. C. Grady , V. WinklerPrins , and A. M. Shortridge . 2013a “Do More Hospital Beds Lead to Higher Hospitalization Rates? A Spatial Examination of Roemer's Law.” PLoS One 8 (2): e54900. 23418432 10.1371/journal.pone.0054900 PMC3572098

[hesr12777-bib-0014] Delamater, P. L. , A. M. Shortridge , and J. P. Messina . 2013b “Regional Health Care Planning: A Methodology to Cluster Facilities Using Community Utilization Patterns.” BMC Health Services Research 13: 333. 23964905 10.1186/1472-6963-13-333 PMC3766152

[hesr12777-bib-0016] Epstein, A. J. 2001 “The Role of Public Clinics in Preventable Hospitalizations among Vulnerable Populations.” Health Services Research 36: 405–20. 11409820 PMC1089231

[hesr12777-bib-0017] Falster, M. O. , L. R. Jorm , K. A. Douglas , F. M. Blyth , R. F. Elliott , and A. H. Leyland . 2015 “Sociodemographic and Health Characteristics, Rather Than Primary Care Supply, Are Major Drivers of Geographic Variation in Preventable Hospitalizations in Australia.” Medical Care 53 (5): 436–45. 25793270 10.1097/MLR.0000000000000342 PMC4396734

[hesr12777-bib-0018] Fiorentini, G. , E. Iezzi , M. Lippi Bruni , and C. Ugolini . 2011 “Incentives in Primary Care and Their Impact on Potentially Avoidable Hospital Admissions.” European Journal of Health Economics 12 (4): 297–309. 20424882 10.1007/s10198-010-0230-x

[hesr12777-bib-0019] Fisher, E. S. , J. E. Wennberg , T. A. Stukel , J. S. Skinner , S. M. Sharp , J. L. Freeman , and A. M. Gittelsohn . 2000 “Associations among Hospital Capacity, Utilization, and Mortality of US Medicare Beneficiaries, Controlling for Sociodemographic Factors.” Health Services Research 34 (6): 1351–62. 10654835 PMC1089085

[hesr12777-bib-0020] Fisher, E. S. , S. M. Shortell , S. A. Kreindler , A. D. Van Citters , and B. K. Larson . 2012 “A Framework for Evaluating the Formation, Implementation, and Performance of Accountable Care Organizations.” Health Affairs (Millwood) 31 (11): 2368–78. 10.1377/hlthaff.2012.0544 23129666

[hesr12777-bib-0021] Garnick, D. W. , H. S. Luft , J. C. Robinson , and J. Tetreault . 1987 “Appropriate Measures of Hospital Market Areas.” Health Services Research 22 (1): 69–89. 3570813 PMC1065423

[hesr12777-bib-0022] Gilmour, S. J. 2010 “Identification of Hospital Catchment Areas Using Clustering: An Example from the NHS.” Health Services Research 45 (2): 497–513. 20050933 10.1111/j.1475-6773.2009.01069.x PMC2838157

[hesr12777-bib-0024] Hu, Y. , F. Wang , and I. M. Xierali . 2018 “Automated Delineation of Hospital Service Areas and Hospital Referral Regions by Modularity Optimization.” Health Services Research 53 (1): 236–55. 10.1111/1475-6773.12616 27861822 PMC5785331

[hesr12777-bib-0025] Kilaru, A. S. , D. J. Wiebe , D. N. Karp , J. Love , M. J. Kallan , and B. G. Carr . 2015 “Do Hospital Service Areas and Hospital Referral Regions Define Discrete Health Care Populations?” Medical Care 53 (6): 510–16. 25961661 10.1097/MLR.0000000000000356

[hesr12777-bib-0026] Klauss, G. , L. Staub , M. Widmer , and A. Busato . 2005 “Hospital Service Areas — A New Tool for Health Care Planning in Switzerland.” BMC Health Services Research 5: 33. 15882463 10.1186/1472-6963-5-33 PMC1131901

[hesr12777-bib-0027] Krakauer, H. , I. Jacoby , M. Millman , and J. E. Lukomnik . 1996 “Physician Impact on Hospital Admission and on Mortality Rates in the Medicare Population.” Health Services Research 31 (2): 191–211. 8675439 PMC1070113

[hesr12777-bib-0028] Kruzikas, D. T. , H. J. Jiang , D. Remus , M. L. Barrett , R. M. Coffey , and R. Andrews . 2004 “Preventable Hospitalisations: A Window Into Primary and Preventive Care, 2000.” HCUP Fact Book No 5; AHRQ Publication No 04‐0056. Rockville, MD: Agency for Healthcare Research and Quality; 2004.

[hesr12777-bib-0029] Landon, B. E. , J. P. Onnela , N. L. Keating , M. L. Barnett , S. Paul , A. J. O'Malley , T. Keegan , and N. A. Christakis . 2013 “Using Administrative Data to Identify Naturally Occurring Networks of Physicians.” Medical Care 51 (8): 715–21. 23807593 10.1097/MLR.0b013e3182977991 PMC3723338

[hesr12777-bib-0030] Larsen, K. , and J. Merlo . 2005 “Appropriate Assessment of Neighborhood Effects on Individual Health: Integrating Random and Fixed Effects in Multilevel Logistic Regression.” American Journal of Epidemiology 161 (1): 81–8. 15615918 10.1093/aje/kwi017

[hesr12777-bib-0031] Leckie, G . 2013 “Multiple Membership Multilevel Models ‐ Concepts.” *LEMMA VLE* Module 13, 1‐61. Available at http://www.bristol.ac.uk/cmm/learning/course.html

[hesr12777-bib-0032] Lewis, V. A. , A. B. McClurg , J. Smith , E. S. Fisher , and J. P. Bynum . 2013 “Attributing Patients to Accountable Care Organizations: Performance Year Approach Aligns Stakeholders’ Interests.” Health Affairs (Millwood) 32 (3): 587–95. 10.1377/hlthaff.2012.0489 PMC4230294 23459739

[hesr12777-bib-0033] Leyland, A. H. , and F. A. Boddy . 1998 “League Tables and Acute Myocardial Infarction.” Lancet 351 (9102): 555–8. 9492774 10.1016/S0140-6736(97)09362-8

[hesr12777-bib-0034] Mazumdar, S. , X. Feng , P. Konings , I. McRae , and F. Girosi . 2014 “A Brief Report on Primary Care Service Area Catchment Geographies in New South Wales Australia.” International Journal of Health Geographics 13 (1): 38. 25292210 10.1186/1476-072X-13-38 PMC4197238

[hesr12777-bib-0036] Merlo, J. , B. Chaix , H. Ohlsson , A. Beckman , K. Johnell , P. Hjerpe , L. Rastam , and K. Larsen . 2006 “A Brief Conceptual Tutorial of Multilevel Analysis in Social Epidemiology: Using Measures of Clustering in Multilevel Logistic Regression to Investigate Contextual Phenomena.” Journal of Epidemiology and Community Health 60 (4): 290–7. 16537344 10.1136/jech.2004.029454 PMC2566165

[hesr12777-bib-0037] Mobley, L. R. , E. Root , L. Anselin , N. Lozano‐Gracia , and J. Koschinsky . 2006 “Spatial Analysis of Elderly Access to Primary Care Services.” International Journal of Health Geographics 5: 19. 16700904 10.1186/1476-072X-5-19 PMC1482683

[hesr12777-bib-0038] NHPA . 2015 Healthy Communities: Potentially Preventable Hospitalisations in 2013–14. Sydney: National Health Performance Authority.

[hesr12777-bib-0039] NSW Health . 2010 NSW Health Services Comparison Data Book 2008/2009. North Sydney, NSW: Demand and Performance Evaluation, NSW Health.

[hesr12777-bib-0040] O'Cathain, A. , E. Knowles , R. Maheswaran , J. Turner , E. Hirst , S. Goodacre , T. Pearson , and J. Nicholl . 2013 “Hospital Characteristics Affecting Potentially Avoidable Emergency Admissions: National Ecological Study.” Health Services Management Research 26 (4): 110–8. 25595008 10.1177/0951484814525357

[hesr12777-bib-0041] Schuurman, N. , R. S. Fiedler , S. C. Grzybowski , and D. Grund . 2006 “Defining Rational Hospital Catchments for Non‐Urban Areas Based on Travel‐Time.” International Journal of Health Geographics 5 (1): 43. 17018146 10.1186/1476-072X-5-43 PMC1617091

[hesr12777-bib-0042] Shwartz, M. , E. A. Pekoz , A. Labonte , J. Heineke , and J. D. Restuccia . 2011 “Bringing Responsibility for Small Area Variations in Hospitalization Rates Back to the Hospital: The Propensity to Hospitalize Index and a Test of the Roemer's Law.” Medical Care 49 (12): 1062–7. 22002646 10.1097/MLR.0b013e3182353907

[hesr12777-bib-0043] Stukel, T. A. , R. H. Glazier , S. E. Schultz , J. Guan , B. M. Zagorski , P. Gozdyra , and D. A. Henry . 2013 “Multispecialty Physician Networks in Ontario.” Open Medicine 7 (2): e40–55. 24348884 PMC3863751

[hesr12777-bib-0044] Wennberg, J. E. , M. McAndrew Cooper , J. D. Birkmeyer , K. K. Bronner , T. A. Bubolz , D. E. Campbell , E. S. Fisher , G. T. O'Connor , J. F. Poage , S. M. Sharp , J. S. Skinner , T. A. Stukel , and D. Wennberg . 1999 The Dartmouth Atlas of Health Care in the United States: Dartmouth Medical School.

[hesr12777-bib-0045] Zhan, C. , M. R. Miller , H. Wong , and G. S. Meyer . 2004 “The Effects of HMO Penetration on Preventable Hospitalizations.” Health Services Research 39 (2): 345–61. 15032958 10.1111/j.1475-6773.2004.00231.x PMC1361011

