# Supplementary material for: Using Weighted Hospital Service Area Networks to Explore Variation in Preventable Hospitalization
Source: Health Serv Res. 2017 Sep 22;53(Suppl Suppl 1):3148–69. doi: 10.1111/1475-6773.12777 (PMC6056604; doi:10.1111/1475-6773.12777)
Supplement: Supplementary file 2 — Table S1: Codes for Identifying Preventable Hospitalizations. Table S2: Incidence Rate Ratios (IRRs) from Multilevel Models with Higher‐Level Units as Either Hospitals in Weighted Hospital Service Area Networks (Weighted‐HSAN) or Hospital Service Areas (HSAs). Table S3: Demographic Distribution of Study Participants and Number of Hospitalizations to Major Public Hospitals. Table S4: Reduction in Deviance Information Criterion (DIC) Statistic From a Single‐Level Age‐ and Sex‐Adjusted Model, Structuring Persons in Various Higher‐Level Units and Sequentially Adjusting for (1) Age and Sex, (2) Further Personal Sociodemographic and Health Characteristics, and (3) Hospital Bed Occupancy. Table S5: Incidence Rate Ratio (IRR) for Preventable Hospitalization from a 10% Increase in Average Hospital Bed Occupancy Rate, from Models* with Higher‐Level Units as Either Hospitals in Weighted Hospital Service Area Networks (Weighted‐HSAN) or Hospital Service Areas (HSAs), and Statistical Local Areas (SLAs). Table S6: Proportional Change in Between‐Hospital, HSA, or SLA Level Variance after Sequentially Adjusting for (1) Age and Sex, (2) Further Personal Sociodemographic and Health Characteristics, and (3) Hospital Bed Occupancy. Figure S1: Correlation between Hospital‐Level Characteristics from Patient Populations Constructed Using a Hospital Service Area (HSA) and Weighted Hospital Service Area Network (Weighted‐HSAN). Figure S2: Model Specification of Two‐Level Multiple‐Membership Multilevel Model, with Persons Structured within a Weighted Hospital Service Area Network (Weighted‐HSAN), Adjusted for Personal Sociodemographic and Health Characteristics, as Well as Average Hospital Bed Occupancy Rate (Continuous Variable, as a 10% Increase). [file HESR-53-3148-s002.pdf]

Table S1

Codes for identifying preventable hospitalizations

Category	ICD-10-AM diagnosis and procedure codes
Chronic	
Angina	I20, I24.0, I24.8, I24.9 as principal diagnosis only, exclude cases with procedure codes not in blocks [1820] to [2016]
Asthma	J45, J46 as principal diagnosis only
Chronic obstructive pulmonary disease (COPD)	J20, J41, J42, J43, J44, J47 as principal diagnosis only, J20 only with additional diagnoses of J41, J42, J43,J44, J47
Congestive cardiac failure	I50, I11.0, J81 as principal diagnosis only, exclude cases with the following procedure codes: 33172-00, 35304-00, 35305-00, 35310-02, 35310-00, 38281-11, 38281-07, 38278-01, 38278-00, 38281-02, 38281-01, 38281-00, 38256-00, 38278-03, 38284-00, 38284-02, 38521-09, 38270-01, 38456-19, 38456-15, 38456-12, 38456-11, 38456-10, 38456-07, 38456-01, 38470-00, 38475-00, 38480-02, 38480-01, 38480-00, 38488-06, 38488-04, 38489-04, 38488-02, 38489-03, 38487-00, 38489-02, 38488-00, 38489-00, 38490-00, 38493-00, 38497-04, 38497-03, 38497-02, 38497-01, 38497-00, 38500-00, 38503-00, 38505-00, 38521-04, 38606-00, 38612-00, 38615-00, 38653-00, 38700-02, 38700-00, 38739-00, 38742-02, 38742-00, 38745-00, 38751-02, 38751-00, 38757-02, 38757-01, 38757-00, 90204-00, 90205-00, 90219-00, 90224-00, 90214-00, 90214-02.
Diabetes complications	E10–E14.9 as principal diagnoses, and E10–E14.9 as additional diagnoses where the principal diagnosis was: hypersmolarity (E87.0), acidosis (E87.2), transient ischaemic attack (G45), nerve disorders and neuropathies (G50–G64), cataracts and lens disorders (H25–H28), retinal disorders (H30–H36), glaucoma (H40–H42), myocardial infarction (I21–I22), other coronary heart diseases (I20, I23–I25), heart failure (I50), stroke and sequelae (I60–I64, I69.0–I69.4), peripheral vascular disease (I70–I74), gingivitis and periodontal disease (K05), kidney diseases including end-stage renal disease (N00–N29), and renal dialysis (Z49)
Hypertension	I10, I11.9 as principal diagnosis only, exclude cases with procedure codes according to the list of procedures excluded from the Congestive cardiac failure category above.
Iron deficiency anaemia	D50.1, D50.8, D50.9 as principal diagnosis only.
Nutritional deficiencies	E40, E41, E42, E43, E55.0, E64.3 as principal diagnosis only.
Rheumatic heart disease	I00 to I09 as principal diagnosis only. (Note: includes acute rheumatic fever)
Acute	
Appendicitis with generalised peritonitis	K35.0 in any diagnosis field
Cellulitis	L03, L04, L08, L88, L98.0, L98.3 as principal diagnosis only, exclude cases with any procedure except those in blocks 1820 to 2016 or if procedure is 30216-02, 30676-00, 30223-02, 30064-00, 34527-01, 34527-00, 90661-00 and this is the only listed procedure
Convulsions and epilepsy	G40, G41, O15, R56 as principal diagnosis only
Dehydration and gastroenteritis	A09.9, E86, K52.2, K52.8, K52.9 as principal diagnosis only.
Dental conditions	K02, K03, K04, K05, K06, K08, K09.8, K09.9, K12, K13 as principal diagnosis only.
Ear, nose and throat infections	H66, H67, J02, J03, J06, J31.2 as principal diagnosis only.
Gangrene	R02 in any diagnosis field
Pelvic inflammatory disease	N70, N73, N74 as principal diagnosis only.
Perforated/bleeding ulcer	K25.0, K25.1, K25.2, K25.4, K25.5, K25.6, K26.0, K26.1, K26.2, K26.4, K26.5, K26.6, K27.0, K27.1, K27.2, K27.4, K27.5, K27.6, K28.0, K28.1, K28.2, K28.4, K28.5, K28.6 as principal diagnosis only.
Pyelonephritis	N10, N11, N12, N13.6, N39.0 as principal diagnosis only.
Vaccine-preventable	
Influenza and pneumonia	J10, J11, J13, J14, J15.3, J15.4, J15.7, J15.9, J16.8, J18.1, J18.8 in any diagnosis field, excludes cases with additional diagnosis of D57 (sickle-cell disorders) and people under 2 months
Other vaccine-preventable conditions	A35, A36, A37, A80, B05, B06, B16.1, B16.9, B18.0, B18.1, B26, G00.0, M01.4 in any diagnosis field

Transfers and type change separations were considered continuation of the same episode of care.

Table S2

Incidence rate ratios (IRRs) from multilevel models with higher-level units as either hospitals in weighted hospital service area networks (weighted-HSAN) or hospital service areas (HSA).

	Weighted-HSAN model		HSA model	
	IRR	(95% CIs)	IRR	(95% CIs)
Hospital bed occupancy (per 10% increase)				
Average across weighted-HSAN	1.01	(0.96 - 1.07)	-	-
Primary hospital of HSA	-	-	1.00	(0.96 - 1.04)
Age				
45-54 years	1.00	(ref)	1.00	(ref)
55-64 years	1.20	(1.15 - 1.26)	1.19	(1.14 - 1.26)
65-74 years	1.68	(1.60 - 1.77)	1.66	(1.57 - 1.76)
75-84 years	2.49	(2.36 - 2.62)	2.45	(2.32 - 2.60)
85+ years	3.29	(3.09 - 3.50)	3.23	(3.02 - 3.46)
Gender				
Male	1.00	(ref)	1.00	(ref)
Female	0.71	(0.69 - 0.73)	0.70	(0.69 - 0.72)
Highest education				
Did not complete high school	1.00	(ref)	1.00	(ref)
High school or equivalent	0.94	(0.91 - 0.96)	0.93	(0.91 - 0.96)
University or higher	0.87	(0.83 - 0.91)	0.86	(0.82 - 0.90)
Unknown/missing	1.13	(1.06 - 1.20)	1.13	(1.06 - 1.20)
Partnership status				
Married or partnered	1.00	(ref)	1.00	(ref)
Single	1.18	(1.13 - 1.24)	1.17	(1.12 - 1.23)
Widowed or separated	1.16	(1.13 - 1.19)	1.16	(1.13 - 1.19)
Unknown/missing	1.26	(1.11 - 1.43)	1.26	(1.11 - 1.43)
Household income				
<$10,000	1.00	(ref)	1.00	(ref)
$10,000 - $29,999	0.88	(0.85 - 0.92)	0.88	(0.85 - 0.92)
$30,000 - $49,999	0.80	(0.76 - 0.85)	0.80	(0.76 - 0.85)
$50,000 - $69,999	0.75	(0.70 - 0.81)	0.75	(0.70 - 0.80)
$70,000 or more	0.65	(0.60 - 0.70)	0.64	(0.60 - 0.69)
Rather not say	0.92	(0.88 - 0.97)	0.92	(0.88 - 0.96)
Unknown/missing	1.13	(1.07 - 1.19)	1.12	(1.07 - 1.18)
Employment status				
Not working	1.00	(ref)	1.00	(ref)
Part time	0.81	(0.77 - 0.85)	0.81	(0.77 - 0.85)
Full time	0.82	(0.78 - 0.87)	0.82	(0.78 - 0.87)
Unknown/missing	0.92	(0.84 - 1.01)	0.92	(0.84 - 1.01)
Language spoken at home				
English only	1.00	(ref)	1.00	(ref)
Other	0.91	(0.87 - 0.95)	0.90	(0.87 - 0.94)
Health insurance status				
None	1.00	(ref)	1.00	(ref)
Private (extras)	1.04	(0.99 - 1.08)	1.04	(1.00 - 1.09)
Private (no extras)	1.28	(1.20 - 1.35)	1.28	(1.20 - 1.35)
DVA health care	1.62	(1.56 - 1.67)	1.63	(1.57 - 1.68)
Health care card	1.51	(1.46 - 1.57)	1.52	(1.46 - 1.58)
Number of people can depend on				
0 people	1.00	(ref)	1.00	(ref)
1-4 people	1.07	(1.02 - 1.13)	1.07	(1.02 - 1.12)
5-10 people	1.05	(1.00 - 1.10)	1.04	(0.99 - 1.10)
11+ people	1.18	(1.11 - 1.25)	1.18	(1.11 - 1.25)
Unknown/missing	1.13	(1.05 - 1.20)	1.12	(1.05 - 1.20)

	Weighted-HSAN model		HSA model	
	IRR	(95% CIs)	IRR	(95% CIs)
Health behaviours [a]				
No positive behaviours	0.88	(0.78 - 0.99)	0.87	(0.78 - 0.98)
1 positive behaviour	1.00	(ref)	1.00	(ref)
2 positive behaviours	0.88	(0.84 - 0.91)	0.87	(0.84 - 0.91)
3 positive behaviours	0.77	(0.74 - 0.80)	0.76	(0.73 - 0.80)
4 positive behaviours	0.75	(0.71 - 0.79)	0.75	(0.71 - 0.79)
Body Mass Index				
Underweight	1.14	(1.10 - 1.19)	1.14	(1.09 - 1.19)
Healthy weight	1.00	(ref)	1.00	(ref)
Overweight	0.94	(0.91 - 0.97)	0.94	(0.91 - 0.97)
Obese	1.00	(0.96 - 1.03)	1.00	(0.96 - 1.03)
Unknown/missing	1.22	(1.10 - 1.34)	1.22	(1.10 - 1.34)
Self-rated health				
Excellent	1.00	(ref)	1.00	(ref)
Very good	1.23	(1.13 - 1.33)	1.22	(1.14 - 1.31)
Good	1.64	(1.51 - 1.78)	1.63	(1.52 - 1.75)
Fair	2.64	(2.42 - 2.88)	2.62	(2.43 - 2.82)
Poor	4.18	(3.81 - 4.60)	4.15	(3.81 - 4.51)
Unknown/missing	2.35	(2.13 - 2.58)	2.33	(2.14 - 2.54)
Number of morbidities [b]				
None	1.00	(ref)	1.00	(ref)
1 morbidity	1.33	(1.28 - 1.38)	1.33	(1.28 - 1.38)
2 morbidities	2.01	(1.93 - 2.09)	2.01	(1.93 - 2.09)
3+ morbidities	2.74	(2.62 - 2.86)	2.74	(2.63 - 2.86)
Functional limitations [c]				
No limitation	1.00	(ref)	1.00	(ref)
Minor limitation	1.02	(0.95 - 1.09)	1.02	(0.95 - 1.09)
Mild limitation	1.24	(1.17 - 1.32)	1.24	(1.17 - 1.32)
Moderate limitation	1.55	(1.46 - 1.65)	1.55	(1.47 - 1.64)
Severe limitation	2.35	(2.21 - 2.50)	2.36	(2.22 - 2.50)
Unknown/missing	1.70	(1.60 - 1.81)	1.70	(1.60 - 1.81)
Psychological distress [d]				
Low distress	1.00	(ref)	1.00	(ref)
Moderate distress	1.02	(0.98 - 1.05)	1.02	(0.98 - 1.05)
High distress	0.95	(0.91 - 1.00)	0.95	(0.90 - 1.00)
Very high distress	0.99	(0.93 - 1.05)	0.98	(0.92 - 1.05)
Unknown/missing	1.12	(1.06 - 1.19)	1.12	(1.06 - 1.19)
Variance				
Weighted-HSAN / HSA (SE)	0.132	(0.032)	0.059	(0.012)
Units of analysis				
Level 1 (people)	266,762	-	266,762	-
Level 2 (hospitals/HSAs)	79	-	72	-

[a] Healthy behaviours, of non-smoking status, safe level of alcohol consumption (<14 drinks per week), at least 2.5 hours of intensity-weighted physical activity per week, and meeting dietary guidelines for daily fruit (2 serves) and vegetable (5 serves) consumption

[b] Of self-reported heart disease, high blood pressure, stroke, diabetes, blood clot, asthma, Parkinson's disease, and any cancer except skin cancer.

[c] Measured using the Medical Outcome Study physical functioning scale.

[d] Measured using the K10 scale.

Table S3

Demographic distribution of study participants and number of hospitalizations to major public hospitals.

	Study population		Number of hospitalizations*			
			All cause		'Preventable'	
	N	% of N	n	% of N	N	% of N
Total (N)	266,762	100	267,032	100	26,728	100
Age						
45-64	163,596	61.3	94,112	35.2	7,651	28.6
65-84	94,913	35.6	154,959	58.0	16,099	60.2
85+	8,253	3.1	17,961	6.7	2,978	11.1
Sex						
Male	123,740	46.4	148,985	55.8	14,678	54.9
Female	143,022	53.6	118,047	44.2	12,050	45.1
Multimorbidity†						
No conditions	108,978	40.9	54,567	20.4	4,488	16.8
1 condition	93,358	35.0	88,140	33.0	7,261	27.2
2 conditions	44,697	16.8	69,834	26.2	7,905	29.6
3+ conditions	19,729	7.4	54,491	20.4	7,074	26.5
Self-rated health						
Excellent/very good	133,871	50.2	58,636	22.0	4,965	18.6
Good	86,928	32.6	88,475	33.1	8,467	31.7
Fair / poor	36,552	13.7	103,923	38.9	11,524	43.1
Missing/unknown	9,411	3.5	15,998	6.0	1,772	6.6

* Hospitalizations to public hospitals, from participants' time of study entry (between 2006-2009) to death or end of linked data (end 2011), whichever came first (average of 3.7 years of follow-up).

† Self-reported conditions, of heart disease, high blood pressure, stroke, diabetes, blood clot, asthma, Parkinson's disease, and any cancer except skin cancer.

Table S4

Reduction in deviance information criterion (DIC) statistic from a single-level age and sex adjusted model, structuring persons in various higher-level units and sequentially adjusting for (1) age and sex, (2) further personal socio-demographic and health characteristics, and (3) hospital bed occupancy.

Higher-level unit(s) of multilevel model	DIC		
	Age and sex	+ socio-demographic and health	+ hospital bed occupancy
No random intercept	0*	24,598	-
Weighted-HSAN	2286	25,467	25,468
HSA	2130	25,544	25,542
SLA	4020	27,028	-
Both weighted-HSAN and SLA	4366	27,262	27,262
Both HSA and SLA	4287	27,254	27,257

*referent category for reduction, DIC value of 185,452

Table S5

Incidence rate ratio (IRR) for preventable hospitalization from a 10% increase in average hospital bed occupancy rate, from models* with higher-level units as either hospitals in weighted hospital service area networks (weighted-HSAN) or hospital service areas (HSA), and statistical local areas (SLA).

Higher-level unit(s) of multilevel model	Incidence rate ratio	
	IRR	(95% CIs)
Weighted-hospital service area network (HSAN) †	1.01	(0.96 – 1.07)
Hospital service area (HSA) ‡	1.00	(0.96 – 1.04)
Both weighted-HSAN and statistical local area (SLA) §	0.98	(0.90 – 1.07)
Both HSA and SLA¶	1.02	(0.96 – 1.08)

* Multilevel Poisson models, adjusted for socio-demographic and health characteristics of study participants.

† Two-level multiple membership multilevel model

‡ Two-level multilevel model

§ Three-level cross-classified multiple membership multilevel model

¶ Three-level cross-classified multilevel model

Table S6

Proportional change in between-hospital, HSA or SLA level variance after sequentially adjusting for (1) age and sex, (2) further personal socio-demographic and health characteristics, and (3) hospital bed occupancy.

Higher-level unit(s) of multilevel model	Variance parameter (and SE of variance)			Proportional change in variance parameter from prior model		
	(1) Age and sex	(2) + socio-demographic and health	(3) + hospital bed occupancy	(1)	(2)	(3)
**Two level models**						
Weighted-HSAN	0.367 (0.084)	0.130 (0.032)	0.132 (0.032)	-	64.6%	-1.5%
HSA	0.105 (0.020)	0.059 (0.012)	0.059 (0.012)	-	43.8%	0.0%
SLA	0.325 (0.042)	0.291 (0.039)	-	-	10.5%	-
**Three level models**						
Weighted-HSAN and SLA						
Weighted-HSAN	0.393 (0.096)	0.234 (0.061)	0.230 (0.062)	-	40.5%	1.7%
SLA	0.316 (0.046)	0.270 (0.040)	0.273 (0.041)	-	14.6%	-1.1%
HSA and SLA						
HSA	0.091 (0.023)	0.089 (0.022)	0.091 (0.022)	-	2.2%	-2.2%
SLA	0.282 (0.040)	0.230 (0.033)	0.231 (0.033)	-	18.4%	-0.4%

Figure S1

Correlation between hospital-level characteristics from patient populations constructed using a hospital service area (HSA) and weighted-hospital service area network (weighted-HSAN)

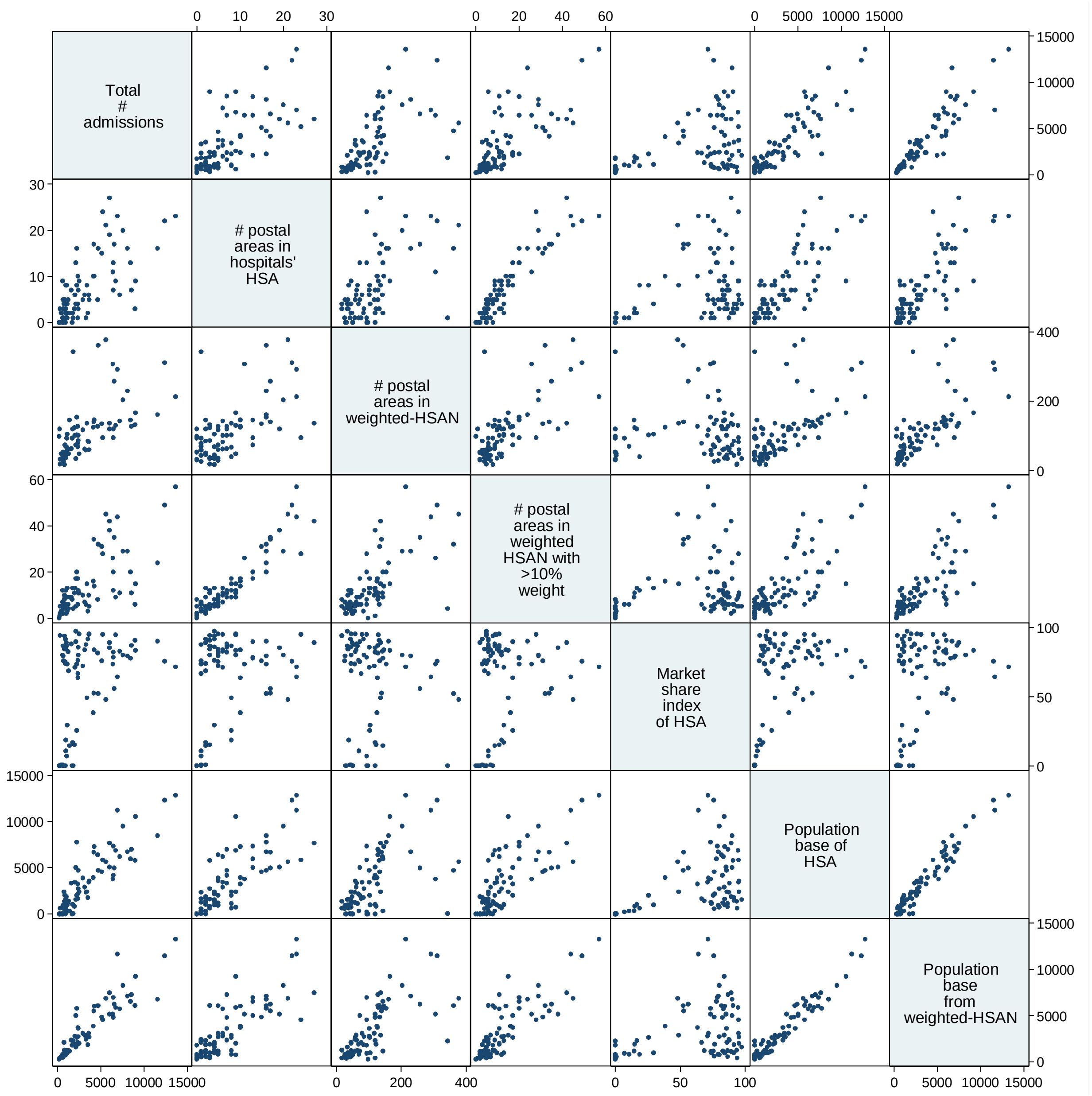

Figure S2

Model specification of two-level multiple membership multilevel model, with persons structured within a weighted hospital service area network (weighted-HSAN), adjusted for personal socio-demographic and health characteristics, as well as average hospital bed occupancy rate (continuous variable, as a 10% increase).

$Y_{ij} \sim Poisson(\pi_i)$

$$\begin{aligned}
\log(\pi_i) = \text{offset}_i &+ \beta_0 + \beta_1\text{age}(55-64) + \beta_2\text{age}(65-74) + \beta_3\text{age}(75-84) + \beta_4\text{age}(84+) \\
&+ \beta_5\text{gender(female)} + \beta_6\text{education(high school)} + \beta_7\text{education(university)} \\
&+ \beta_8\text{education(unknown)} + \beta_9\text{partnership(single)} + \beta_{10}\text{partnership(separated)} \\
&+ \beta_{11}\text{partnership(unknown)} + \beta_{12}\text{income}(\$10-29\text{k}) + \beta_{13}\text{income}(\$30-50\text{k}) \\
&+ \beta_{14}\text{income}(\$50-70\text{k}) + \beta_{15}\text{income}(\$70\text{k}+) + \beta_{16}\text{income(not say)} \\
&+ \beta_{17}\text{income(unknown)} + \beta_{18}\text{employment(part time)} + \beta_{19}\text{employment(full time)} \\
&+ \beta_{20}\text{employment(unknown)} + \beta_{21}\text{language(other)} + \beta_{22}\text{insurance(private} - \text{extras)} \\
&+ \beta_{23}\text{insurance(private)} + \beta_{24}\text{insurance(DVA)} + \beta_{25}\text{insurance(health care card)} \\
&+ \beta_{26}\text{socialsupport}(1-4\text{ people}) + \beta_{27}\text{socialsupport}(5-10\text{people}) \\
&+ \beta_{28}\text{socialsupport}(11+\text{people}) + \beta_{29}\text{socialsupport(unknown)} \\
&+ \beta_{30}\text{healthbehaviours}(1) + \beta_{31}\text{healthbehaviours}(2) + \beta_{32}\text{healthbehaviours}(3) \\
&+ \beta_{33}\text{healthbehaviours}(4) + \beta_{34}\text{BMI(healthy)} + \beta_{35}\text{BMI(overweight)} + \beta_{36}\text{BMI(obese)} \\
&+ \beta_{37}\text{BMI(unknown)} + \beta_{38}\text{health(verygood)} + \beta_{39}\text{health(good)} + \beta_{40}\text{health(fair)} \\
&+ \beta_{41}\text{health(poor)} + \beta_{42}\text{health(missing)} + \beta_{43}\text{morbidity}(1) + \beta_{44}\text{morbidity}(2) \\
&+ \beta_{45}\text{morbidity}(3+) + \beta_{46}\text{limitations(minor)} + \beta_{47}\text{limitations(mild)} \\
&+ \beta_{48}\text{limitations(moderate)} + \beta_{49}\text{limitations(severe)} + \beta_{50}\text{limitations(unknown)} \\
&+ \beta_{51}\text{distress(moderate)} + \beta_{52}\text{distress(high)} + \beta_{53}\text{distress(veryhigh)} \\
&+ \beta_{54}\text{distress(unknown)} + \beta_{55}\sum_{j=1}^{79} w_{j.i}^{(2)} bedoccupancy(10\%increase)^{(2)} \\
&+ \sum_{j=1}^{79} w_{j.i}^{(2)} u_j^{(2)}
\end{aligned}$$

Where $u_j^{(2)} \sim N(0, \sigma_u^2)$

With hospital-level weights $\left(0 \le w_{j.i}^{(2)} \le 1\right)$ and $\left(\sum_{j=1}^{79} w_{j.i}^{(2)} = 1 \text{ for all } i\right)$

And offset$_i$ is the logarithm of the follow-up time for individual i.
